# Therapeutic Potential of Capsaicin against Cyclophosphamide-Induced Liver Damage

**DOI:** 10.3390/jcm12030911

**Published:** 2023-01-24

**Authors:** Mohammad Firoz Alam, Ahmed O. Ajeibi, Majed H. Safhi, Ahmad J. A. Alabdly, Saeed Alshahrani, Hina Rashid, Marwa Qadri, Abdulmajeed M. Jali, Saud Alqahtani, Yousra Nomier, Sivakumar S. Moni, Mohammad Khalid, Tarique Anwer

**Affiliations:** 1 Department of Pharmacology and Toxicology, College of Pharmacy, Jazan University, Jazan 45142, Saudi Arabia; 2 Department of Pharmacology, College of Pharmacy, King Khalid University, Abha 61421, Saudi Arabia; 3 Department of Pharmaceutics, College of Pharmacy, Jazan University, Jazan 45142, Saudi Arabia; 4 Department of Pharmacognosy, College of Pharmacy, Prince Sattam Bin Abdulaziz University, Alkharj 11942, Saudi Arabia

**Keywords:** cyclophosphamide, hepatotoxicity, capsaicin, oxidative stress, inflammatory cytokines, histopathology

## Abstract

Cyclophosphamide (CPM) is a classical alkylating agent used in different cancer chemotherapy regimens and is restricted due to severe adverse effects, including hepatotoxicity. Natural or plant-derived antioxidants such as capsaicin were utilized in this study to examine the hepatoprotective benefits against cyclophosphamide-induced hepatotoxicity. The rats were divided into five groups: a normal control group, a toxic group (CPM), an intraperitoneal injection of a single dose of 200 mg/kg b.w. on the fourth day, a pretreated group with two doses of CPS (10 mg and 20 mg/kg b.w.) orally for six consecutive days, and an intraperitoneal administration of 200 mg/kg b.w. on the fourth day of treatment. The fifth group was administered with the highest dose of CPS (20 mg/kg b.w.) orally for six consecutive days. After 24 h of administration of CPS, the rats were anesthetized, blood was collected, and the serum enzyme toxicity was evaluated. After the blood sampling and euthanasia of all the animals, the liver was isolated for further toxicity and histopathological examination. The results revealed that serum liver markers (AST, ALT, ALP, BLI) significantly increased after CPM administration, but were subsequently restored after CPS treatment with both doses. In addition, lipid peroxidation (MDA), inflammatory cytokines (IL-1β, TNF-α), and apoptotic markers (Caspase-3) increased, and antioxidant enzymes (GSH, CAT, SOD) were significantly decreased after CPM administration, and it was re-established by CPS treatment. However, CPS effectively protected against the CPM-induced histopathological architects of liver tissues. In conclusion, CPS attenuates CPM-induced hepatotoxicity via modulating oxidative stress, apoptotic signals, and cytokine pathway. Therefore, CPS could play a significant role as a supplement during the chemotherapy of patients.

## 1. Introduction

Cyclophosphamide (CPM) is a commonly and widely used cancer treatment drug [1,2]. Additionally, it possesses strong immunosuppressive activity and is utilized clinically to treat autoimmune disorders [3]. However, the clinical application of CPM has often been restricted due to its various side effects, including hepatotoxicity [4,5]. The precise mechanism of CPM-induced liver injury is poorly understood. As we know, the liver is the principal detoxifying organ that maintains metabolic homeostasis. Cyclophosphamide is metabolically activated by the hepatic enzyme cytochrome P450 MFO (mixed-function oxidase) and produces the active metabolites acrolein and phosphoramide mustard, which cause oxidative stress in tissue [6,7]. The antineoplastic effects of CPM have been linked to phosphoramide, whereas acrolein has toxic effects such as necrosis, apoptosis, oncosis, and cell death [8,9]. Some experimental investigations suggest that oxidative stress also contributes to hepatotoxicity related to CPM, which is characterized by increased blood markers and changes in liver architecture [10,11,12].

The uses of natural antioxidants have been increased to minimize the side effects of chemotherapeutic drugs in recent years. Despite this trend, very few experimental studies have focused on the prevention of CPM-induced hepatotoxicity. A review of the literature indicates that several prominent natural antioxidants are available, but most of them, including capsaicin, have not been examined.

Capsaicin (CPS) is a well-known active antioxidant compound found in hot peppers that offers significant health benefits such as analgesic, anti-cancer, anti-inflammatory, and antioxidant properties [13]. The anti-inflammatory and antioxidant system controls macrophages’ ability to produce pro-inflammatory mediators and reactive oxygen species [14,15]. Macrophages are white blood cells of the immune system that engulf and digest antigens. Macrophages also have a significant anti-inflammatory function and can reduce the immune response by releasing cytokines. Macrophages that promote inflammation are known as M1 macrophages, whereas macrophages that reduce inflammation and aid in repair are known as M2 macrophages. Thus, CPS could play an important therapeutic role in minimizing the side effects of CPM in liver tissues. Hence, the present study has been designed to explore the potential therapeutic effects of capsaicin on CPM-induced hepatic injury in rats.

## 2. Materials and Methods

### 2.1. Chemicals, Kits, and Drugs

Cyclophosphamide, Capsaicin, 1-chloro-2,4-dinitrobenzene (CDNB), 5-5′-ditho-bis-2-nitrobenzoicacid (DTNB), and Thiobarbituric acid were procured from Sigma Aldrich Co. 3300 S 2nd St. Louis, MO, 63118, USA. Serum marker assay kits (AST, ALT, ALP, Bilirubin) were procured from Randox Lab Ltd., 55 Diamond Road, Crumlin, County Antrim, BT29 4QY, U. K, and cytokines (IL-1β and TNFα) and caspase 3 were bought from Abcam Discovery Drive, Cambridge Biomedical Campus, Cambridge, CB2 0AX, UK. Before further analysis, all chemicals, drugs, and diagnostic kits were stored at the specified temperature.

### 2.2. Experimental Design

**Figure 1:**
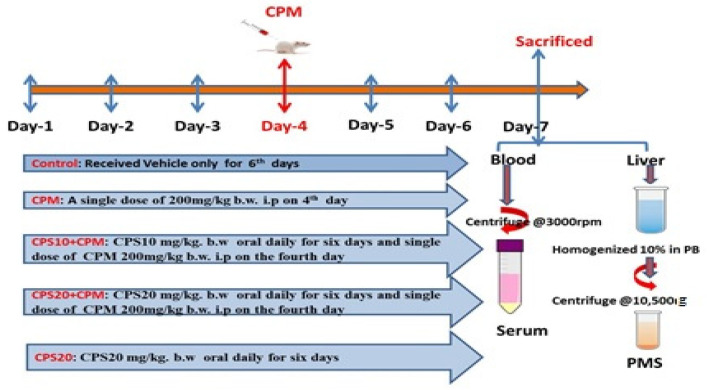
Experimental design and animal sampling preparation.

The planned experiments were carried out with the approval of Jazan University’s Scientific Research Ethics Committee (No.REC41/1-033). The Medical Research Center of Jazan University provided the Wistar albino rats for this study. Before starting the experiment, rats were housed for a week to adapt to the environment. The rats were maintained under normal environmental conditions, including temperature (22 ± 2 °C), humidity (45–55%), photoperiod (12 h light/12 h dark), and constant ventilation. The rats were free to eat and drink, and food and water were replenished daily. In the present study, the rats (180–220 g) were randomly divided into five groups, comprising six rats each. The details of the groups are as follows: the control group (CNT) received a single intraperitoneal injection of normal saline and oral water for six days: The cyclophosphamide (CPM) group received a single intraperitoneal dose of 200 mg/kg b.w. (represented as CPM200) on the fourth day; the capsaicin (CPS) control group received 20 mg/kg b.w. (represented as CPS20) orally for six days. The rats were pre-treated with Capsaicin orally at 10 mg/kg b.w. [16] (represented as CPS10) and 20 mg/kg b.w. [13] (represented as CPS20) for six days followed by the administration of a single dosage of CPM (200 mg/kg) [17] intraperitoneally on the fourth day of the experiment, as seen in the diagram below (Figure 1).

After seven days of continuous capsaicin administration, the rats were fasted, given water, and sacrificed under anesthesia (87 mg/kg b.w. ketamine and 13 mg/kg b.w. xylazine) [18]. In addition, blood samples were collected from the rats by the retro-orbital plexus method and placed in test tubes without anticoagulants to determine serum biomarkers. Each group’s liver tissue was isolated to determine the physical appearance, liver weight, abnormality count, and histopathology. For the assay of antioxidant enzymes and inflammatory mediators, the liver tissue was homogenized. Using phosphate buffer (0.01 M and pH 7.4) under a homogenizer, kidney tissue was used to create the 10% homogenate. Afterwards, the 10% homogenate was centrifuged at 3000 rpm for 10 min at 4 °C, and the upper layer, named the supernatant, was collected for use in the lipid peroxidation test. In a similar manner, 10% homogenate was centrifuged at 10,500 g for 10 min at 4 °C, and the upper layer, named post the mitochondrial supernatant (PMS), was collected to test for other antioxidant enzymes. For further research, the isolated liver sample was immediately stored at −20 °C.

### 2.3. Assessment of Liver Function

The serums were isolated from the blood sample by centrifuging for 10 min at 3000 rpm. Then, the serum samples were analyzed to determine AST (aspartate transaminase), ALT (alanine transaminase), ALP (alkaline phosphatase), and BLI (bilirubin) with the help of the standard procedure of the Randox^TM^ assay kit.

### 2.4. Assessment of Oxidative Stress

Liver tissue samples were used to analyze the LPO (lipid peroxidation), GSH (reduced glutathione), CAT (catalase), and superoxide dismutase (SOD) as described by Utley et al. (1967), Jallow (1974), Claiborne (1985), and Stevens et al. (2000), respectively [19,20,21,22]. In brief, these markers were evaluated with minor changes as per Alam (2018a) [23]. The assay of LPO was carried out using a UV-1601, Shimadzu, Japan, spectrophotometer, at a wavelength of 535 nm. The obtained value was expressed in nmol TBARS formed/h/mg protein using a molar extinction coefficient (MEC) of 1.56 × 10^5^ M^−1^ cm^−1^. The absorbance of GSH was determined at 412 nm, and the value was expressed as the amount of DTNB conjugate formed per mg protein using 13.6 × 10^3^ M^−1^ cm^−1^. The CAT absorbance was monitored at 240 nm, and the value was expressed as H_2_O_2_ consumed/min/mg protein using MEC 43.6 × 10^3^ M^−1^ cm^−1^. The SOD was estimated by the monitoring of the auto-oxidation of (-) epinephrine at 480 nm, and the value was expressed as nmole(-) epinephrine protected using MEC 4.02 × 10^3^ M^−1^ cm^−1^.

### 2.5. Assessment of Cytokines and Apoptosis Markers

An ELISA assay was performed for the investigation of IL-1β and TNFα using a Bio-Tek Elisa reader (ELX800, Vermont 05404-0998, USA) and the Abcam manufacturer’s guidelines at 450 nm. The apoptosis marker caspase-3 was analyzed as per the Abcam standard protocol using a microplate reader at 405 nm. The data were analyzed according to the manufacturer’s scheme.

### 2.6. Histopathological Assessment

The separated rat liver was preserved with a 10% formaldehyde solution before being embedded in paraffin as part of the standard histology procedures [24]. These embedded samples were sectioned by a Leica microtome into 5 µm-thick sections and stained with hematoxylin-eosin (H&E). Finally, the H&E-stained sample was observed under a light microscope (Leica) at 40× magnification. The markers of inflammation, apoptosis, and regeneration were scored using a 0–4 scale. In brief, centrilobular apoptosis was graded as grade 0, which indicates normal without any changes, grade 1 indicated centrilobular apoptosis around the central vein (one-third of acinar zone 3), grade 2 indicated centrilobular apoptosis (acinar zone 3 to acinar zone 2), occasional connecting, and massive necrosis, grade 3 indicated connecting and submassive necrosis involving <50% of the liver section, and grade 4 indicated massive coagulation and apoptosis >50% of the liver section. Based on the size and frequency, inflammatory lesions were graded as follows: 0 for normal, 1 for minimal, 2 for mild, 3 for moderate, and 4 for severe. Similarly, liver regeneration was evaluated on a 0–4 scale depending on the frequency of hepatocyte mitotic figures identified in the hepatocyte region. Whereas a score of 0 reflects no mitotic figures, a score of 1 represents 1–2 mitotic figures, a score of 2 represents 3–4 mitotic figures, and a score of 4 represents >7 mitotic figures. As a result, the total injury score was 0 for normal, 1 for minimum, 2 for mild, 3 for moderate, and 4 for severe [25].

### 2.7. Protein Assessment

Protein levels in the liver tissue were determined using the Lowry et al. (1951) method, with bovine serum albumin serving as a reference standard [26].

### 2.8. Statistical Analysis

The data derived from the various experiments were analyzed using the latest GraphPad Prism Software 9, and the value was expressed as the mean ± sd of six rats. Analysis of variance (ANOVA) was used to identify significant group differences, followed by the Tukey–Kramer test. If the *p*-value was less than 0.05, the observed differences between the experimental groups were deemed to be statistically significant.

## 3. Results

### 3.1. Effect of Capsaicin on Liver Function and Morphology

The control group exhibited a normal level of AST, ALT, ALP, and bilirubin, whereas the CPM group exhibited a significantly (p< 0.0001) higher level of these markers. On the other hand, there was a considerable decrease in these markers after treatment with CPS10 and CPS20 compared to the CPM200 group. No substantial (*p* > 0.05) changes were seen in the CPS20 group associated with the normal control (Table 1). The effects of capsaicin pretreatment on body weight, liver weight, and their ratio against cyclophosphamide intoxication in rats are shown in Table 2. CPM-induced rat intoxication showed a significant increase in liver weight as compared to the normal control. CPS was increased by 4.80% when the percent of liver weight to body weight ratio was increased. Pretreatment with CPS significantly attenuated the liver weight and body weight ratio by 2.86%. Thus, CPS minimizes the body weight, liver weight, and liver weight ratio levels of rats (Table 2).

### 3.2. Effect of Capsaicin on Oxidative Stress

**Figure 2:**
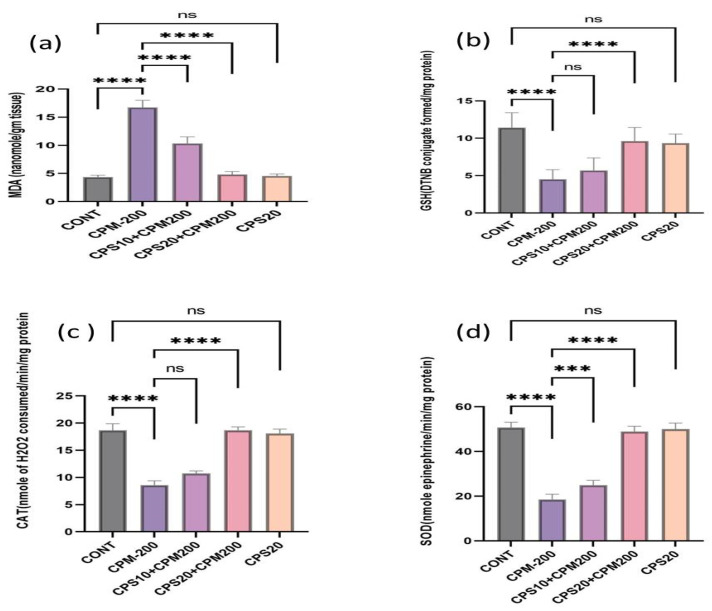
(**a**–**d**) represents the therapeutic effects of capsaicin on oxidative stress markers (MDA, GSH, CAT, and SOD). Data represented the mean value ± SD (*n* = 6). Figure 2a represents the effects of the capsaicin on MDA and found significant improvement **** *p* < 0.0001 CPS vs. CPM, ^ns^ *p* > 0.05 CPS vs. CNT; Figure 2b represents the effects of capsaicin on GSH and found **** *p* < 0.0001 CPM vs. CONT, **** *p* < 0.0001 CPS vs. CPM, ^ns^ *p* > 0.05 CPS vs. CONT; Figure 2c represents the effect of capsaicin on catalase (CAT) and found **** *p* < 0.0001 CPM vs. CONT, **** *p* < 0.0001 CPS vs. CPM, ^ns^ *p* > 0.05 CPS vs. CONT; Figure 2d represents the effect of capsaicin on superoxide dismutase (SOD) and found **** *p* < 0.0001 CPM vs. CONT, *** *p* < 0.001 CPS10 vs. CPM, **** *p* < 0.0001 CPS vs. CPM, ^ns^ *p* > 0.05 CPS vs. CONT.

The CPM group rats expressed an increase in hepatic lipid peroxidation (MDA) and a reduction in glutathione (GSH) content. In contrast, the treatment groups CPS10 + CPM200 and CPS20 + CPM200 displayed a significant protective role against CPM-induced hepatic toxicity. It was noticed that the GSH content improved after the treatment with CPS10 and CPS20, and the MDA level was reduced in contrast to the CPM-induced group. In addition, other antioxidant enzymes (catalase, superoxide dismutase) were also depleted after CPM administration and improved after treatment with both doses of CPS. There were no significant (p > 0.05) differences between CPS20 and the control (Figure 2a–d).

### 3.3. Effect of Capsaicin on Inflammatory Cytokine and Apoptotic Markers

**Figure 3:**
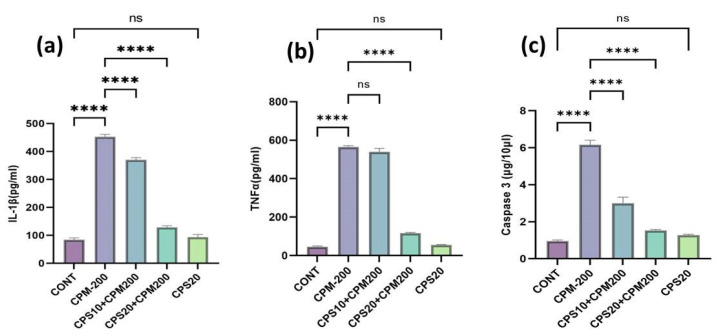
(**a**–**c**) represents the therapeutic effects of Capsaicin on inflammatory cytokines and apoptotic markers. Figure 3a represents the effects of capsaicin on IL-1β and found significant improvement **** *p* < 0.0001 CPS vs. CPM; Figure 3b represents the effects of capsaicin on TNFα and found significant improvement **** *p* < 0.0001 CPS20 vs. CPM but not effective with the lowest dose ^ns^ *p* > 0.05 CPS vs. CPM; Figure 3c represents the effects of capsaicin on caspase-3 and found significant improvement **** *p* < 0.0001 CPS vs. CPM; no Significant (^ns^ *p* > 0.05) changes were seen in CPS20 vs. CONT.

The higher dose of CPS was more effective and significant in the case of inflammatory cytokines than lower doses of CPS. IL-1β exhibited significant (*p* < 0.001) elevation in the liver of the CPM-administered group compared to the normal control group. It reduced the IL-1β after treatment with CPS, and both doses of CPS were more effective and significant (*p* < 0.0001), as represented in Figure 3a. CPM administration significantly (*p* < 0.0001) increased pro-inflammatory cytokines such as TNF-a, as shown in Figure 3b, compared to the normal controls. Oral supplementation with CPS remarkably reduced TNFα in the treatment groups compared to the CPM-administered group. CPM administration significantly (*p* < 0.0001) increased apoptotic markers such as Caspase-3, as represented in Figure 3c, compared to the normal controls. On the other hand, oral supplementation with CPS (10 and 20 mg) showed a significant (*p* < 0.001) reduction in the level of caspase-3 in both CPS-treated groups compared to the CPM-administered groups. The higher dose of CPS was more effective and significant than the lower doses of CPS. There are no significant differences between the highest amount of CPS and the standard control.

### 3.4. Effects of Capsaicin on Liver Histology

**Figure 4:**
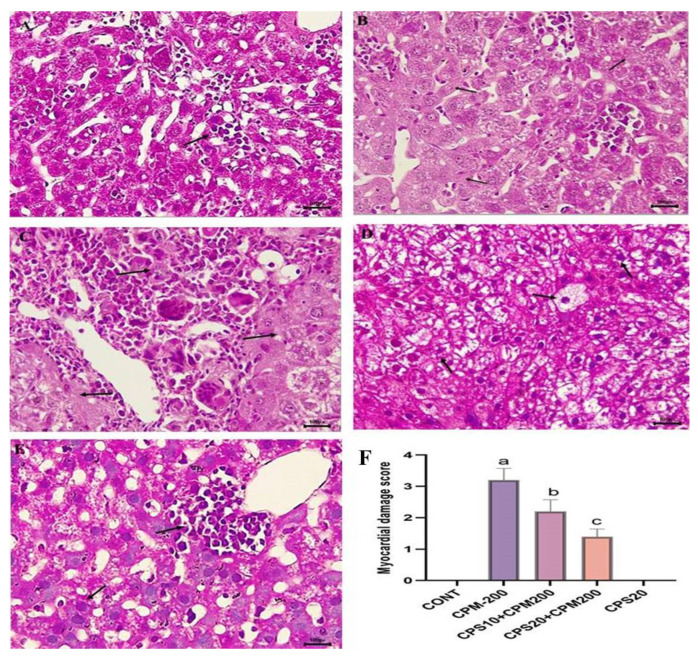
(**A**–**F**) represent the therapeutic effects of capsaicin on liver histology against CPM-induced hepatotoxicity. All the injury scores were represented in a bar graph with *p* values. Hematoxylin and Eosin (H&E)-staining indicates (**A**), normal control (CONT) without any abnormality indicated by arrow—score = 0; (**B**), CPM intoxication with massive degeneration and necrosis in focal areas marked by arrow—score = 3.5 (^a^ *p* < 0.001 vs. CONT); (**C**), CPS10 + CPM200 is capsaicin treatment with mild improvement in hepatic histology marked by arrow—score = 2 (^b^ *p* > 0.05 vs. CPM200); (**D**) CPS20 + CPM200 is capsaicin treatment with significant improvement in hepatic histology marked by arrow—score = 1 (^c^ *p* < 0.001 vs. CPM200); and (**E**) CPS20 no significant changes were found in liver tissue and seems to be the normal control marked by arrow—score = 0 (^b^ *p* > 0.05 vs. CONT). (**F**) All hepatocytes damage score were represented in bar graph and found significant injury in CPM-200 vs control (^a^ *p* < 0.001); treatment with CPS10 were not effective and not significant (^b^ *p* > 0.05) effective vs CPM200 but treatment with CPS20 showed significant (^c^ *p* < 0.001 vs. CPM200) improvement and no injury in CPS20 vs CONT.

CPM administration showed histological changes such as focal areas with massive coagulation, massive degeneration, severe inflammation, and necrosis. The injury score was extreme (score 4) as shown in Figure 4B. The pretreatment with the lowest dose of CPS10 revealed a marked improvement in the liver histological structure, and the liver damage score was mild (score = 2) as shown in Figure 4C. The pretreatment with the highest dose (CPS20) significantly improved the histological structure, and the minimal injury score was (score = 1), as shown in Figure 4D. In contrast, the H&E-stained liver section of the normal control showed a regular structure of hepatocytes without any alteration, as represented in Figure 4A, with an injury score of 0. On the other hand, following the pretreatment with the highest dose of CPS20 alone, no significant structural changes were observed in liver tissue, Figure 4E, with an injury score of 0.

## 4. Discussion

Cyclophosphamide is used as a chemotherapeutic drug for cancer patient survival, but its hepatotoxicity cannot be ignored because it always has complications and challenges. CPM undergoes metabolic changes by liver enzymes and forms 4-dyroxychlophosphamide, further breaking into two cytotoxic metabolites called acrolein and phosphoramide mustard. Phosphoramide mustard can be dephosphoramidated to create nornitrogen mustard, which has an alkylating action [27,28]. Cyclophosphamide-induced hepatotoxicity is well recognized and reported [29]. Current chemotherapeutic strategies must be supplemented with natural antioxidants to prevent hepatotoxicity. In this study, capsaicin is investigated to determine the potential protective mechanisms associated with inflammatory cytokine apoptosis and oxidative stress against CPM-induced hepatotoxicity. In the present study, CPM administration causes liver damage due to ROS, which leads to oxidative damage in vital organs [30]. The generation of reactive oxygen species (ROS) is a significant factor in liver damage and the onset of hepatic fibrogenesis [31]. Reactive oxygen species (ROS) are low-molecular-weight molecules that are highly reactive and short-lived. Free radicals such as the superoxide anion (O_2_^•^) and the hydroxyl radical (OH^•^) are produced from oxygen, along with non-radical molecules such as hydrogen peroxide (H_2_O_2_) [32]. Therefore, CPM administration induces liver injury, as seen in elevated liver enzymes such as AST, ALT, ALP, and bilirubin. These enzymes are important markers of the liver function test. Thus, an elevated marker is a direct indicator of hepatocyte injury. In the present study, these markers were significantly higher in the CPM-administered group. CPS treatment repairs this damage by decreasing these levels due to its particular antioxidant properties. The biological antioxidant substances may protect cells and tissues from the harmful effects of ROS and other free radicals caused by CPM [33,34,35]. Animal research conducted over the past few decades has demonstrated that CPM-induced hepatotoxicity is related to free radical oxidative stress. Oxidative stress mediates a wide range of hepatic impairments due to free radicals. CPM administration resulted in lipid peroxidation; the MDA (malondialdehyde) level was increased compared to the standard control, and the antioxidant enzymes (GSH, CAT, and SOD) were significantly reduced. Several investigations have indicated that free radicals play an important role in CPM-induced liver pathology [36,37]. Once a hepatic injury is induced by CPM, this further leads to the leaching out of markers of liver damage from hepatocytes. CPM administration also causes histopathological changes by producing inflammation, congestion, epithelial degeneration, and vacuolization in liver tissue. In support of this finding, many researchers have reported cases of extensive swelling and the sinusoidal narrowing of liver tissue in cyclophosphamide-treated rats [38,39].

In addition, inflammatory cytokines also play an important role in understanding the vital role of pathogenesis and the molecular mechanism of CPM-induced hepatotoxicity. Oxidative stress is favorably associated with inflammation [40]. Inflammation, apoptosis, and fibrosis are associated with the increased expression, activation, and nuclear translocation of NF-kB p65. Thus, inhibiting the p65 subunit could be a method for CPS to release inflammatory cytokines and interleukin. CPM is well recognized as an activator of NF-kB p65 that leads to the transcription of inflammatory cytokines (TNFα), interleukins (ILs), and fibrosis [41,42,43,44]. NF-kB induces apoptotic cell death by increasing the transcriptional expression of fatty acid synthetase (FAS) ligands in response to reactive oxygen species (ROS). FAS ligands interact with the FAS on neighboring hepatocytes, causing caspase-9 activation and the subsequent activation of additional caspases, including caspase-3, resulting in apoptotic hepatocyte cell death [45]. Caspase-3 is a crucial protein in the apoptotic cascade, cleaving many cellular proteins and triggering the death of old, damaged, and self-reactive cells. Inflammatory cytokines such as tumor necrosis factor (TNFα) and interleukin-1 (IL-1β) are essential in the fundamental inflammatory process. TNF-alpha is a cytokine that regulates inflammation, cell death, and proliferation by activating several intracellular pathways. Whereas TNFα contributes to the development of hepatotoxicity in the liver, it also contributes to the restoration of functional liver mass by stimulating hepatocyte proliferation and liver regeneration [46]. Moreover, although TNFα has a protective effect on liver regeneration, the role of TNFα during liver injury remains a matter of debate. An earlier report showed that the concentration of TNFα influences the actions of the protein. High doses of TNFα have been shown to exacerbate lipopolysaccharide-induced liver damage. The level of TNFα remained unchanged during acetaminophen (APAP)-induced hepatotoxicity, indicating that the drug was having a protective impact [47]. The precursor protein for IL-1β is digested by caspase-1, also known as the IL-1β converting enzyme. Other serine proteases also process the IL-1β precursor. An earlier report suggested that primary murine hepatocytes are sensitized to Fas ligand (FasL)-induced caspase-3/-7 activation by the pro-inflammatory cytokine IL-1 β [48].

The present study demonstrated a substantial increase in TNFα, IL1β, and Caspase-3 following CPM administration compared to controls. Alqahtani and Mahmoud (2016) also reported that CPM induced hepatocyte apoptosis; it found a significant increase in caspase-3 expression and protein levels [49]. Caglayan et al. (2018) also reported that CPM (200 mg/kg b.w.) stimulated the apoptotic and autophagy pathways by increasing the expression of cysteine aspartate-specific protease-3 caspase-3 [50]. The outcomes of the current investigation are comparable to those indicated before. Capsaicin (CPS) is an intense component of hot red pepper, widely used for antioxidant, anti-inflammatory, and anti-apoptotic action in a cell by inhibiting the synthesis of ROS and inflammatory cytokines. It inhibits the NF-kB pathway, which plays a vital role in tumorigenesis and negatively impacts on cell cycle homeostasis. It also helps to reduce the production of ROS by increasing antioxidant enzymes. In this study, CPS displayed better ameliorating effects on liver redox status in the treated group, and it was more significant in higher doses of CPS20 than in lower doses of CPS10 compared to the toxic group (CPM). No significant changes were seen between the highest doses of capsaicin CPS20 and the usual control group. Thus, in current studies, CPS plays a detoxifying role by improving the antioxidant enzymes GSH, CAT, and SOD.

The above biochemical findings were also confirmed by histopathological changes and the amelioration effect of CPS along with CPM administration. The sections of liver obtained from CPM-treated rats revealed extensive degeneration, significant inflammatory cell filtration, and necrosis in the majority of the hepatic parenchyma. Conversely, no histological changes were seen in the tissue sections of the group treated with CPS alone. Interestingly, the liver sections of both treated groups nearly resembled those of the normal control group.

## 5. Conclusions

Cyclophosphamide (CPM) produces liver damage by disrupting the antioxidant system by producing ROS, generating lipid peroxidation, activating inflammatory cytokines, and expressing caspase-3. Capsaicin (CPS) confers an appealing hepatoprotective effect against cyclophosphamide-induced hepatotoxicity via diminishing ROS generation, improving the antioxidant system, and inhibiting inflammatory and apoptotic expression. Thus, CPS may contribute a novel and targeted hepatoprotective supplement for chemotherapeutic drugs to minimize the adverse effects of CPM.

## Figures and Tables

**Table 1:** Therapeutic effect of Capsaicin on serum markers against cyclophosphamide-induced hepatotoxicity.

Groups	AST (U/L)	ALT (U/L)	ALP(U/L)	BLI (mg/dL)
CONT	45.5 ± 3.27	27.83 ± 4.26	113.33 ± 5.82	1.225 ± 0.27
CPM-200	97.16 ± 4.71 ^a^	77.66 ± 2.88 ^a^	383.66 ± 4.50 ^a^	2.4 ± 0.32 ^a^
CPS-20	46.83 ± 5.64 ^b^	34.33 ± 4.13 ^b^	123.33 ± 4.55 ^b^	1.30 ± 0.09 ^b^
CPS10 + CPM200	85.16 ± 7.01 ^c^	62.33 ± 5.01 ^c^	257.5 ± 4.76 ^c^	2.25 ± 0.19 ^c^
CPS20 + CPM200	57 ± 4.20 ^d^	38.66 ± 4.55 ^d^	128.5 ± 5.47 ^d^	1.32 ± 0.15 ^d^

Therapeutic action of capsaicin on serum markers against CPM-induced hepatotoxicity. Values are presented as mean ± sd (*n* = 6), ^a^ *p* < 0.0001 vs. CONT, ^b^ *p* > 0.05 vs. CONT, ^c,d^ *p* < 0.001 vs. CPM200; Abbreviations: AST—aspartate transaminase; ALT—alanine transaminase; ALP—alkaline phosphatase; and BLI—bilirubin.

**Table 2:** Effect of Capsaicin on liver weight, body weight and their ratio against cyclophosphamide intoxication in rats.

Groups	Body Weight (g)	Liver Weight (g)	LW/BW(%)
CONT	222.60 ± 3.78	5.41 ± 0.11	2.43 ± 0.09
CPM-200	187.60 ± 11.34 ^a^	8.98 ± 0.06 ^a^	4.80 ± 0.30 ^a^
CPS20	218.60 ± 2.30 ^ns^	5.25 ± 0.13 ^ns^	2.40 ± 0.07 ^ns^
CPS10 + CPM200	197.20 ± 6.90 ^b^	8.48 ± 0.40 ^b^	4.30 ± 0.25 ^b^
CPS20 + CPM200	216.80 ± 4.21 ^c^	6.21 ± 0.266 ^c^	2.86 ± 0.10 ^c^

Values are presented as mean ± sd (*n* = 6), ^a^ significant difference in body weight, liver weight, liver weight and body weight ratio (^a^ *p* < 0.0001 vs. CONT, ^ns^ *p* > 0.05 vs. CONT, ^b,c^ *p* < 0.001 vs. CPM200). Abbreviations: CONT—control; CPM—cyclophosphamide; CPS—capsaicin; LW—liver weight; and BW—body weight.

## Data Availability

The authors confirm that the data are contained within the article.
